# Geo-Mapping of the Spatial Accessibility to Public Oral Health Facilities among Schoolchildren in Selangor, Malaysia

**DOI:** 10.3390/healthcare11101405

**Published:** 2023-05-12

**Authors:** Nurul Izzati Mohamad Ali, Jamaludin Marhazlinda

**Affiliations:** 1 Department of Community Oral Health & Clinical Prevention, Faculty of Dentistry, Universiti Malaya, Kuala Lumpur 50603, Malaysia; 2 Oral Health Program, Ministry of Health, Putrajaya 62590, Malaysia; 3 Community Oral Health Research Group, Faculty of Dentistry, Universiti Malaya, Kuala Lumpur 50603, Malaysia

**Keywords:** geo-mapping, geographic information system, dental health service, health service accessibility, health inequalities, travel distance, travel time, schoolchildren

## Abstract

Spatial accessibility to health services influences service utilisation and eventually impacts the disease burden. This cross-sectional study analysed the spatial accessibility of schoolchildren to public oral health facilities and school dental services (SDS) and vice versa in Selangor. Overlay and proximity analysis from geo-mapping software was employed to map the primary dental clinics with SDS, the public schools, and the proximity between primary dental clinics with SDS and public schools by travelling distance (5 km, 10 km, 20 km) and travelling time (15 min, 30 min). Over half of the schoolchildren in Selangor are within 5 km of accessibility to primary dental clinics and SDS teams. Meanwhile, nearly half of the primary and secondary schools, particularly in rural areas, are located within a more than 5 km service area of public oral health facilities. The SDS teams have a travel burden of more than 20 km to the public schools in Selangor’s northern and north-western districts of large geographical areas. Simultaneously, most public primary and secondary schools are within 15 min of driving time from primary dental clinics. Geo-mapping highlights the inequalities in spatial accessibility to public oral health facilities with SDS among schoolchildren in Selangor. It is time to prioritise the resources, SDS, and preventive programmes to reduce inequalities in oral health accessibility among schoolchildren in Selangor.

## 1. Introduction

While many countries’ oral health status has improved significantly over the last decade, oral health status and treatment needs are unevenly distributed, particularly among children [1,2]. In addition, societal disparities in oral health are still prevalent in both developing and developed nations. Individuals at the lower end of the socioeconomic spectrum face a more significant burden of poor health issues than those at the higher end [3]. It shows that improved oral health has not equally benefited all children.

In terms of oral health related quality of life (OHRQoL) among children, untreated dental caries and its consequences considerably impact their OHRQoL. Research shows dental caries continues to be the most detrimental dental condition to children’s quality of life [4]. Furthermore, this condition may contribute to high school absenteeism and poor performance [5]. Therefore, in the purview of oral healthcare for schoolchildren in Malaysia, school dental service (SDS) programme has been at the core of oral services as continuous efforts to improve the oral health of schoolchildren, starting from an early age in all states, including Selangor.

In general, SDS is delivered via outreach mode using the following methods: school dental clinics, mobile dental clinics, and the SDS mobile dental team. The school dental clinics are established in schools with the support of the Ministry of Education, Malaysia. Schools with dental clinic setups provide oral healthcare and treatments not only for schoolchildren but also for teachers and parents. Mobile dental clinics are dental facilities that have been converted from vehicles, such as buses and trailers, to serve remote areas and areas without dental clinics or facilities. At the same time, the SDS mobile dental team composed of dental personnel who travel from one school to school. This team is equipped with portable dental units, which include portable dental chairs and dental instruments that allow more student participation in dental examinations and treatments. The team will provide oral health services by serving in schools with school dental clinics or by delivering via temporary dental clinics. The same mobile dental team is also responsible for delivering oral healthcare to rural areas where dental clinics do not exist. The role of the mobile dental team in SDS is to provide dental examinations, curative treatments based on the treatment needs, and oral health education and promotion. During the visiting period of the mobile dental team to the assigned schools, dental screenings or examinations and necessary treatments will be completed during school hours. Students will receive referral letters for continued care at a nearby public dental clinic only if it is determined that they require complex or specialised treatment [6,7]. Parents will bring their children to the clinic with the referral letter. When the dental visit at a school has been completed, the mobile dental team will move to another school according to the visit schedule outlined by the dental officers’, therapists’, and teachers’ agreements.

The backbone of the SDS is a dental workforce composed of dental officers, dental therapists, dental surgery assistants, and healthcare assistants from the Ministry of Health (MOH), Malaysia. The SDS team is stationed in public dental clinics. The number of the school’s mobile team varies across states in Malaysia and heavily relies on posts provided by the MOH, Malaysia [6]. This programme, delivered through an incremental approach, aims to improve the oral health status of schoolchildren aged seven- to twelve-year-olds and thirteen- to seventeen-year-olds who attended public primary and secondary schools in the country. This programme is proven to provide accessibility to oral healthcare, as reports have shown that most public primary and secondary schools (>90%) have been visited and covered with proper treatments from 2014 to 2019, before the pandemic [7]. Through the SDS program, public schoolchildren in Malaysia are eligible to receive oral health education, dental check-ups, and required treatments at least once a year. Written consent from parents is obtained prior to clinical procedures such as extractions and fillings. The consent from parents is renewed annually, as schools are visited once a year, every year. The SDS programme in Malaysia aligns with global evidence that a school-based oral healthcare initiative is a cost-effective means of promoting long-term oral health [8,9,10]. Thus, the implementation of SDS in Malaysia has consistently emphasised early preventative strategies rather than restorative ones alone, as evidenced by studies of a school-based dental care programme in numerous neighbouring countries, highlighting the importance of oral health promotional activities in school settings [11,12]. This is essential to ensure that schoolchildren are rendered orally fit once they leave school.

Since its inception in 1948 and the introduction of the incremental dental care (IDC) programme in schools (1985), there has been a marked improvement in the oral health status of children and adolescents [13]. Besides the improvement of the oral disease burden, SDS has demonstrated its ability to reduce the inequalities among schoolchildren in Malaysia, particularly in Selangor. For instance, school coverage with completion of dental check-ups and necessary treatments among primary schools across districts in Selangor was 94.5 percent in 2018 [6,7]. These findings showed that SDS effectively reaches a large proportion of schoolchildren as it increases the accessibility of oral healthcare in Selangor. Geographic information system (GIS) has gained appeal among researchers as a tool to assess accessibility to oral healthcare. The increased availability of data sets and improved analytical function of GIS have inspired advances in measuring health accessibility [14,15]. GIS is now extensively used in various countries to analyse market segmentation and network analysis for health access and planning [16,17,18]. Furthermore, the GIS can demonstrate public access and quality of services in the study area, allowing for an understanding of the impact of the location of primary care clinics on urban health outcomes, health-related travel, and service delivery [19,20]. As a result, understanding their accessibility, including the physical locations of health services and their distance from one another, is essential. A recent review of oral health accessibility for Universal Health Coverage (UHC) has indicated that GIS is a powerful tool for integrating spatial and non-spatial factors involving concepts of accessibility [21].

There are a handful of studies on GIS and health accessibility, however, only a few of them concern Malaysia. A study by Bohari et. Al., 2019 used three different distances, 5 km, 10 km, and 20 km, to look into practice distribution of public and private practices for a larger geographical area involving the whole state of Malaysia [22]. This study visualises access to dental practises using Euclidean distance, often known as straight line distance, with buffers of 5 km, 10 km, and 20 km [22,23]. In recent years, researchers have become increasingly interested in calculating spatial accessibility in terms of travel distance and travel time using actual road networks to highlight access to healthcare. The road networks present a better and more accurate depiction of distance, particularly when showing patients’ geographic accessibility [24]. All agreed that this method provided a more meaningful representation of spatial access because the road network accounts for geographic barriers such as rivers, lakes, mountains, and areas without road networks [18,25,26]. In Malaysia, especially in Klang Valley, the primary mode of transportation is by road and private car [27]. A study conducted in 2010 showed that only 17% of trips each day were completed using public transport, and the rest of the 83% were made through private transport [28]. A later study in 2014 by Chuen et al. revealed that travellers, especially in the Klang Valley, preferred to use private cars as their main mode of transportation to commute from one place to another [27].

To date, there has been little to no research on the spatial accessibility of public oral health facilities, specifically SDS, via actual road networks in Malaysia. This research gap warrants an investigation into Selangor’s geographical accessibility for future recommendations on oral healthcare delivery, such as mobile dental teams of the SDS programme or even additional oral healthcare facilities in underserved areas. Furthermore, the findings will serve as a guideline for future research. Due to the unavailability of data from the school dental clinic and mobile dental clinic during this period, this study focused on and assessed the spatial accessibility of public oral healthcare facilities with the SDS mobile team among Selangor’s primary and secondary schoolchildren in terms of travelling distance and time using the actual road network from the clinics to the schools and vice versa.

## 2. Materials and Methods

This was a cross-sectional study investigating the spatial accessibility of public oral health facilities delivering SDS to public primary and secondary schoolchildren relative to the sociodemographic and socioeconomic characteristics of the population of Selangor. It mapped the geographic distribution of public primary and secondary schools and public dental clinics or oral healthcare facilities with SDS mobile dental teams in Selangor. Catchment areas were generated using GIS analysis to visualise health accessibility regarding travel distance and time following the actual road network, calculated from public dental clinics as the centroid to public schools in Selangor. Ethical approval was obtained from the Medical Research & Ethics Committee (MREC) and secured permissions from the Ministry of Health (MOH) Malaysia (NMRR-19-3017-50706).

### 2.1. Study Area

**Figure 1:**
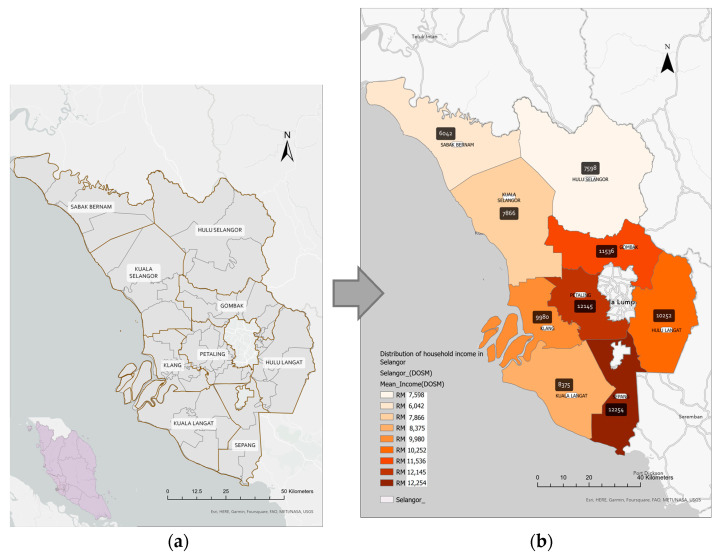
Map of Selangor; (**a**) distribution of nine administrative of Selangor; (**b**) distribution of mean household income by administrative districts in Selangor.

The research was conducted in Selangor Darul Ehsan (Figure 1a), one of the states in Peninsular Malaysia’s central region. Selangor occupies an area of approximately 8000 square km along Peninsular Malaysia’s western coast at the northern outlet of the Straits of Malacca. Selangor is Malaysia’s most prosperous state due to its advantageous geographic location and abundant natural resources. It consists of nine administrative districts. *Gombak, Klang, Petaling, Sepang,* and *Hulu Langat* are five urban districts. The remaining four rural districts are *Kuala Selangor, Kuala Langat, Sabak Bernam,* and *Hulu Selangor*. The capital city of Selangor is *Shah Alam*, which is situated in the *Petaling* district. The Household Income Survey and Basic Amenities Report by State and Administrative District, Selangor, 2019 provided location strata and household gross income distribution by administrative districts in Selangor (Figure 1b). The five urban districts have appeared as districts with a higher household gross income than other districts (Table 1).

### 2.2. Study Population

The study population was all primary and secondary schools and their schoolchildren receiving IDC through SDS in Selangor in 2019. In 2019, 667 primary schools (524,876 students) and 269 secondary schools (340,873 students) received IDC in Selangor. Public oral health facilities include primary dental clinics delivering SDS, hospital- and non-hospital-based specialist clinics, and visiting clinics.

### 2.3. Variables of the Study

#### 2.3.1. Public Oral Health Facilities

The physical address of each public primary dental clinic in Malaysia was gathered from open sources, including the Official Portal of Ministry Health Malaysia, which is downloadable via the website https://www.moh.gov.my/ (accessed on 1 January 2022) [29]. These addresses were cross-checked with the *Taburan Fasiliti dan Perkhidmatan Pergigian* Report, 2019. The latitude and longitude of each address were geocoded in ArcGIS Pro (Version 2.9) software, which converted all the addresses into points on the base map, also provided in the software. Randomly selected samples of 1–2% of all clinics were tested against personal knowledge and web searches via Google Earth, which provides a satellite view of the location.

#### 2.3.2. Public Schools’ Location and Student Enrolment

The dataset of the public school’s location includes school codes, addresses, postcodes, email addresses, coordinates, and students’ enrolment of each school was retrieved from the Educational Planning & Research Division, Ministry of Education, Malaysia. Data was formatted correctly in a comma delimit (csv file) and exported into the software.

#### 2.3.3. State and District Divisions

In a geospatial study, the most important dataset was the spatial component, which includes the area and boundaries of the study area. The state boundaries data was a polygon shapefile downloaded from the ESRI Living Atlas from ArcGIS Online. The shapefile was projected into the WGS 1984 Web Mercator Coordinate System. This projected coordinate system was selected for use throughout the research as it is the most common map projection and matched with the coordinates provided by the Ministry of Education. Therefore, this coordinate system was also considered the most appropriate for the study.

#### 2.3.4. Socioeconomic Characteristics of Selangor

Information on mean household income by administrative districts as a characteristic of the socioeconomic status of Selangor was retrieved from the Department of Statistics Malaysia (DOSM) [30].

#### 2.3.5. Parameters of Travelling Distances and Travelling Time

The parameters used for oral health accessibility were aligned and referred to studies from Tennant and Kruger [31] and Bohari et al., 2019 [22], which utilised buffer zones of 5 km, 10 km, and 20 km distances for oral health accessibility of the catchment service area. As for travel time, 15 and 30 min travel times were referred to a study by Shubayr et al., 2022 [24].

### 2.4. Study Tool

All mapping and geocoding of data used ArcGIS Pro Version 2.9 from ESRI USA to geo-map the spatial accessibility, and the data were tabulated in Excel in Microsoft 365.

### 2.5. Conduct of the Study

All the extracted data were formatted in an Excel sheet and converted to a CSV file prior to data transfer. The file was then exported to the software and integrated with the base map provided by the software. Geocoding of the public dental clinics and school locations was completed using GIS software (ArcGIS Pro Version 2.9, ESRI, Redlands, CA, USA).

#### 2.5.1. Geocoding Approach

All the public dental clinics and school locations were successfully geocoded and included in the study. Due to ethical reasons, the exact schoolchildren’s home addresses are not permissible. Therefore, all schoolchildren were geocoded to school locations. Furthermore, the children in Selangor are enrolled based on the nearest distance of the children to the public schools. Currently, school availability is within a 5-kilometre radius of households in Selangor. In 2019, households with fewer than 5 km from government primary and secondary schools were recorded at 99.3 percent (2016: 96.2%) and 98.6 per cent (2016: 91.5%), respectively [30]. Due to such considerations, public school locations served as a proxy for the location and population density of public schoolchildren in Selangor. Therefore, calculating the accessibility catchment areas was deemed viable and relevant for this study.

#### 2.5.2. Overlay and Proximity Analysis

Using Network Analysis toolset embedded in ArcGIS Pro version 2.9, proximity and overlay analyses were performed to produce the desired map. All the primary dental clinics and primary and secondary schools receiving IDC under the SDS programme, and the base map of Selangor’s state and district boundaries were integrated into one map. Toolset determined geographic accessibility based on the travelling distance and time of the actual road network. Each distance parameter was set to a 5 km, 10 km, and 20 km radius depicted as polygons represented by different colours, with public oral health facilities as the centre point of the calculations. Polygons generated represent the catchment or service coverage. The distributions of school locations, which also served as proxies for the schoolchildren’s location and population density, were overlayed or coincided with the generated polygons. Maps with the schools that were overlayed within the pre-set distance (i.e., represented by coloured polygons) were generated as an image format and information was retrieved and translated into tables. The analysis was repeated for travel time using different parameters, which were 15 min and 30 min. The generated service area was applicable for round-trip or back-to-back distances and times.

## 3. Results

Selangor has 89 public oral health facilities, which include primary care clinics (also known as primary dental clinics), hospital-based specialities (Oral Maxillofacial and Paediatric Dentistry), and non-hospital-based specialities (Orthodontics, Periodontics, Restorative Dentistry). Aside from a standalone dental clinic, public oral health facilities frequently share buildings with other public medical health facilities, such as health clinics and hospitals. Most non-hospital-based specialist clinics coexist with primary dental clinics as they share the same facilities as Primary Healthcare Clinics, except for two standalone orthodontic specialist units in *Klang and Gombak.* In addition, most primary dental clinics were found near each other, especially in urban districts: *Klang, Hulu Langat,* and *Petaling.* Therefore, the public oral health facilities have 89 clinics, including 67 primary dental clinics (inclusive of visiting clinics and UTC), seven hospital-based specialists and two standalone non-hospital-based specialist clinics. At the same time, the other 13 non-hospital-based specialist clinics are attached to the primary dental clinics. Fractions of facilities in Selangor were detailed as follows (Table 2).

**Figure 2:**
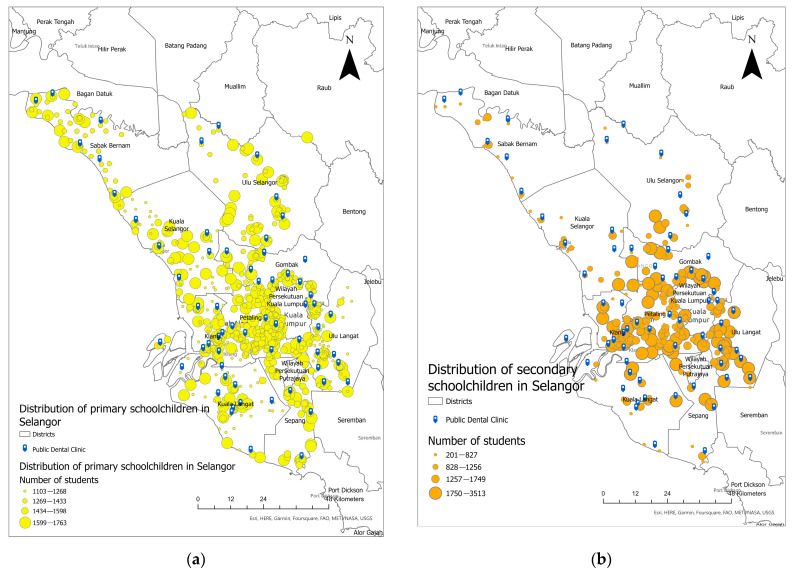
Map of Selangor: (**a**) Distribution of public primary dental clinics and primary schoolchildren of Selangor; (**b**) distribution of public secondary dental clinics and secondary schoolchildren in Selangor. Dot sizes represent the number of schoolchildren. As larger the size, the larger the number of schoolchildren.

Figure 2 shows the distribution of all public oral healthcare facilities/dental clinics and the distribution of primary and secondary schoolchildren geo-coded to school locations, respectively, in Selangor. A dark blue tag represents all primary dental clinics whereas yellow and orange dots represent the distribution of primary and secondary schoolchildren geocoded to school location, respectively. Generally, the primary dental clinics in Selangor were not evenly distributed throughout the state. They were concentrated in urban districts in the centre of Selangor, such as Klang, Hulu Langat, and *Petaling.* Meanwhile, primary dental clinics in the northern and north-western parts of Selangor, such as *Hulu Selangor, Sabak Bernam,* and Kuala Selangor, were much sparser despite their large geographic area. Thus, the overall distribution is not even throughout the state (Figure 2a,b). 

**Figure 3:**
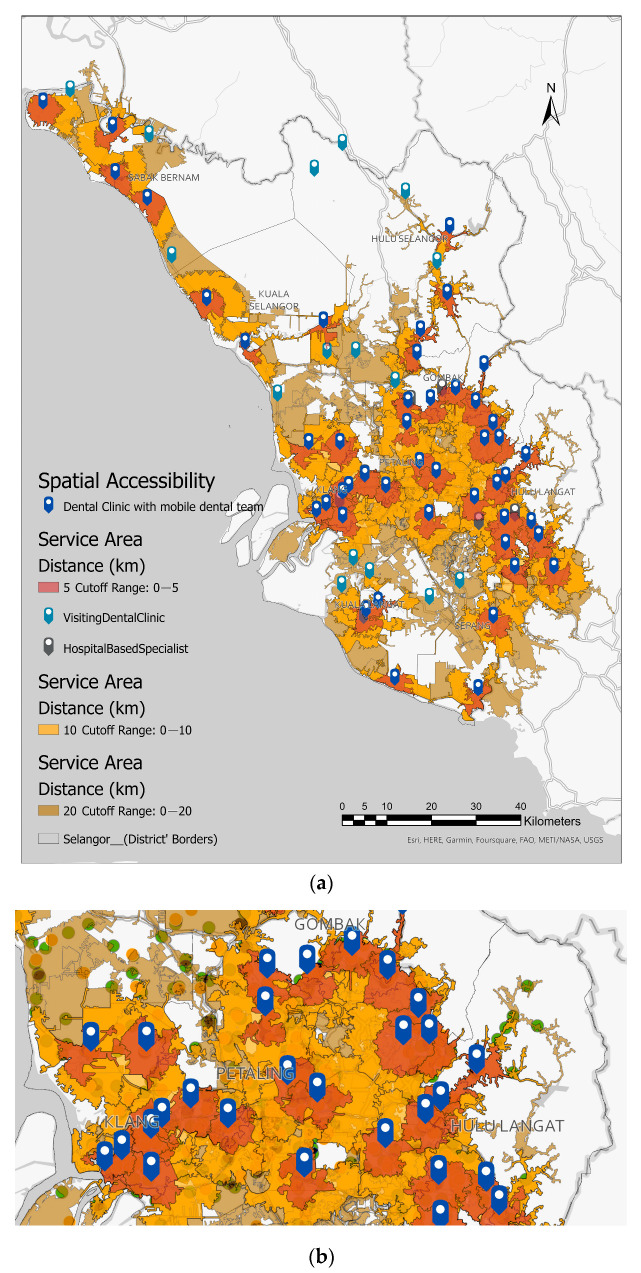
(**a**) The map shows an estimated catchment area of 5 km (represented by a dark brown color), 10 km (a light orange color), and 20 km (represented by a light brown colour) for the primary dental clinic in Selangor. These hierarchical polygons were calculated from primary dental clinics as the centroid using the actual road network for motor vehicles, which includes private vehicles and public transport like buses. (**b**) A magnified subsection of the urban districts of Selangor.

Figure 3 illustrates the estimated catchment or coverage area of 5, 10, and 20 km of primary dental clinics in Selangor. Three different colours of polygons are light red, representing the 5 km catchment area and nearest to public dental clinics. Next is orange, which represents the catchment area of 10 km, and yellow represents the catchment area of 20 km. The map depicts that dental service coverage in urban districts and towards cities in the centre of Selangor were close to each other, as the polygons overlapped and shared the same access road network to nearby clinics (Figure 3b). Despite a larger geographical area in the northern districts of Selangor, which constitute the rural districts, the number of clinics is smaller, and the distributions of catchment areas are more dispersed and far from each other. Slightly more than half of the primary schools (324, 52.61%), accounting for 211,629 students were located outside the public dental clinics’ 5 km catchment or service area (light red areas). Meanwhile, 107 (40%) of secondary schools were located outside a 5 km catchment or coverage area. However, these secondary schools accounted for more than half of the enrollment, indicating that while more than half of the schools are within 5 km, large proportions of student enrolment were outside the 5 km catchment areas. Details are also shown in Table 3 and Table 4.

**Figure 4:**
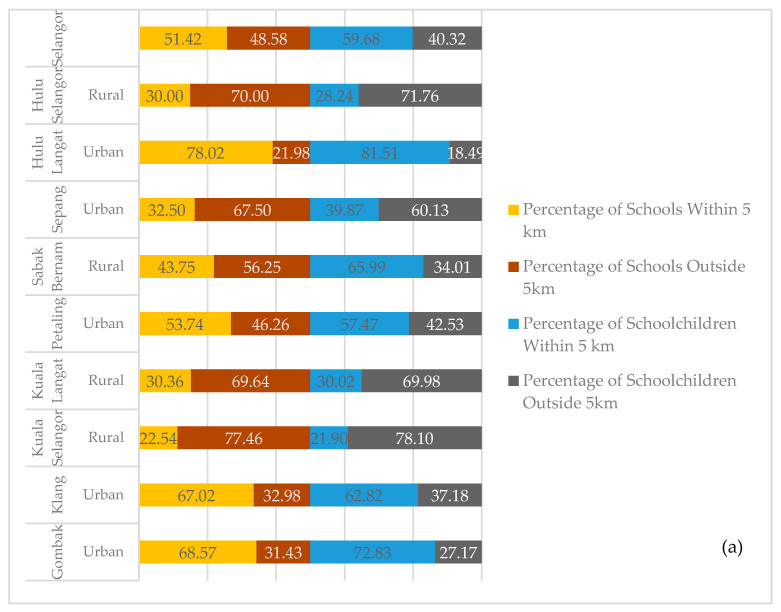

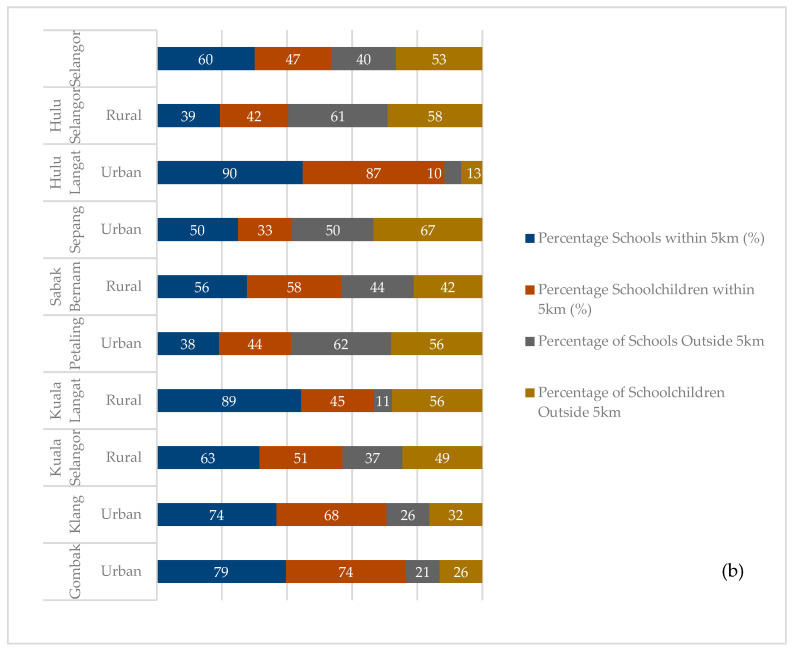
Proportion of (**a**) primary schools and schoolchildren (**b**) secondary schools and schoolchildren in different districts located within travel distance of 5 km and outside of the catchment area of 5 km, including 10 and 20 km in Selangor.

As distance increased, this study found that only 2% of primary and 1% of secondary schools were located outside the 20 km service or catchment area, which involved 11 out of 667 primary schools (599 students) and 3 out of the 269 secondary schools (1170 students). All these schools were in rural districts and among the lowest ranking in terms of the mean administrative districts of Selangor. It indicates a coverage gap of 2% and 1% in primary and secondary schools in Selangor, which suggests that the mobile dental teams in some districts of Selangor travelled more than 20 km to provide dental check-ups and treatments, especially in the districts of *Kuala Selangor*, *Kuala Langat,* and *Hulu Selangor*. Details of travel distances for primary and secondary schoolchildren are shown in Table 3 and Table 4, respectively. Figure 4 denotes the proportion of primary and secondary schools from urban and rural districts that resided within a 5 km catchment area compared with schools located outside 5 km, including schools located within 10 and 20 km of a primary dental clinic.

**Figure 5:**
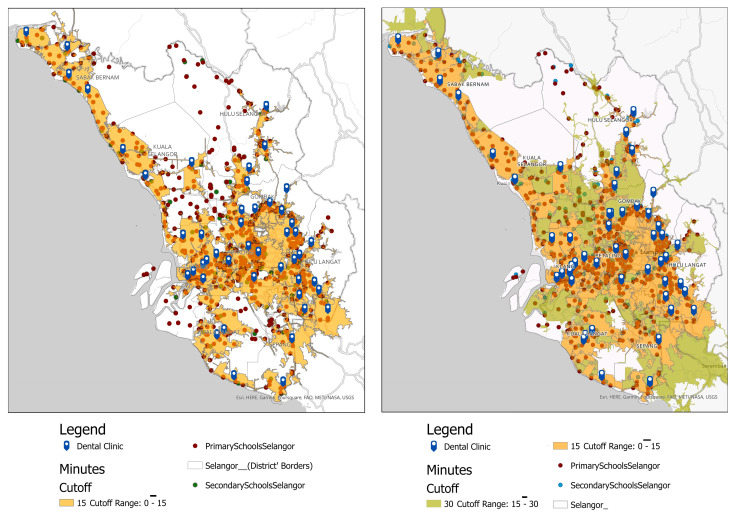
The map shows the catchment area or service coverage of 15 min and 30 min of the primary dental clinic in Selangor.

Figure 5 depicts the travel time catchment area based on 15 min and 30 min drive times for primary and secondary schools in Selangor. On the map, diffused light orange polygons represented the area of 15 min of drive time, whereas blue tags represented primary oral healthcare clinics. These hierarchical polygons were calculated from the primary dental clinic as the centroid using the actual road network for motor vehicles, which includes private vehicles and public transport like buses. There were 23 (3.45%) primary schools (dark red dots) out of 667 primary schools, and 7 out of 267 secondary schools (2.00%) (teal dots) located outside 15 minutes’ drive from the primary oral healthcare facilities. It indicates access to primary dental clinics is within 15 minutes’ drive time. Details of the number of primary and secondary schools situated outside the estimated 15- and 30-min drive areas are shown in Table 5.

## 4. Discussion

The key study findings are about the spatial or geographic accessibility of public schoolchildren to receive oral health services in Selangor, specifically regarding travel distance and time. Our study findings reveal that the SDS teams had to travel more than 5 km to reach almost half of the public schools in Selangor to deliver oral health services. Therefore, if referred for complex treatment, the schoolchildren in nearly half of all public primary schools in Selangor would need to travel more than 5 km to receive primary oral healthcare services. This finding also holds for secondary schoolchildren, with nearly half of all public secondary schools in the state located more than 5 km from primary dental clinics. Findings from the study also illustrated an overlapping of 5 km of service areas between primary dental clinics in urban areas. This demonstrates that schoolchildren living within the service area and the overlapping areas had less access to public dental care within 5 km. Therefore, the schoolchildren could choose from a small handful of accessible public dental clinics nearby, regardless of their respective districts. This is considering that children must register for a school nearby their home, and more than 95% of households in Selangor are within 5 km of schools.

The results of our study can be interpreted with a few limitations. First, the exact location of schoolchildren was geocoded based on school location. The travel distance and travel time generated are estimations of where the schoolchildren lived as they shared the main postcodes of their respective schools. Nevertheless, the majority of students that were geocoded to school locations were considered to live nearby based on DOSM 2020 reports [30]. Since the travel distance and travel time generated were based on the actual road network, the finding is still viable as private cars and public transport like buses are using the same roads to public dental clinics. Subsequently, the present study is limited to public primary and secondary schoolchildren in Selangor, which does not account for private schoolchildren and home-schooled children in Selangor. Including this group would possibly influence the dental caries cluster patterns and the investigation of the accessibility of oral health care services. This study does not assess the travel distance and time using public transport or walking distances. The findings could be more interesting if these two modes of transportation were included, as they offer more valuable insights into accessibility among schoolchildren and other age groups like adults or senior citizens. Future research could also collect additional data on the number of school dental clinics and mobile dental clinics actively providing dental care in Selangor and other Malaysian states. This could be the area of emphasis for rural schools with extremely limited access and long travel times or distances from dental clinics.

In Malaysia, the development of public healthcare centres is heavily reliant on the Five Year Malaysia Plan (MP) [32]. It is a comprehensive blueprint for the socioeconomic development of Malaysia prepared by the Economic Planning Unit (EPU) of the Prime Minister’s Department (PMO) and the Ministry of Finance that is updated every five years. Therefore, new healthcare facilities and any expansions depend highly on this plan and the budgets allocated by the Ministry of Finance, Malaysia [32]. All this may explain the uneven distribution and slower growth of dental clinics in Selangor.

The study markedly found that the SDS teams had a substantially lower travel burden for primary and secondary schoolchildren in urban areas, indicating better accessibility to primary oral health services than their rural counterparts. Study findings also supported the idea that the SDS programme could be expanded to private schools in urban districts as these areas offer more oral healthcare facilities and the mobile dental team has a lower travel burden [33,34,35]. However, more detailed data is required to support the readiness of the SDS programme to expand to private schools. As a result, this study may spark more in-depth research to better understand schoolchildren’s accessibility and the SDS in other states in Malaysia.

On the other hand, the maps presented in this study revealed that the SDS mobile teams in Selangor’s rural districts had to travel more than 20 km to reach Selangor’s primary and secondary public schools to provide oral health care. It also illustrates how the SDS mobile teams increased service utilisation among rural schoolchildren despite needing to travel longer distances than the team that served in urban areas [36]. Other studies concur with our findings that the SDS programme has benefited schoolchildren in remote areas [36]. Our finding also provided valuable insights into the SDS teams’ efforts in rural districts, pointing out the broader coverage they must shoulder due to the uneven distribution of public oral healthcare facilities in Selangor, especially in rural areas. It highlights the importance of workforce planning, specifically the optimal workforces for the SDS teams in rural districts, considering the geographic distance, the number of students that need to be served, and their disease burden [36,37,38,39,40].

The present study also discovered inequality in oral healthcare access among primary and secondary schools across the districts in Selangor. This makes the school location the best proxy for their home location. Schoolchildren who resided in rural and large geographical areas had a higher travel burden in terms of travel distance and time taken to primary oral health clinics and hospitals. Although it is not the only barrier to oral healthcare access, geographic barriers are significant for remote and low-income populations, as they are less likely to own a car and live in areas with limited access to public transportation [31,40,41]. Hence, providing even oral health care access, primarily through the SDS, could be prioritised for schoolchildren in Selangor and Malaysia.

Travel time, as compared to other measurements, accurately represents accessibility for oral healthcare facilities [24]. However, there has yet to be an ideal travel time to healthcare. The study corroborated the previous literature, using 15 and 30 min travel times to healthcare facilities [42]. Other studies have used a range of 5 to 60 min but have categorised the well-served areas as taking less than or equal to 30 min and the underserved regions as taking more than 30 min [24]. Nonetheless, each study has a similar cut-off regarding an acceptable travel time of 30 min to healthcare facilities. In this study, more than 90 per cent of the public primary and secondary schoolchildren had access to oral healthcare within 15 min of driving time, with primary and secondary schoolchildren in urban districts having a much lower travel burden to access primary oral healthcare clinics than their other counterparts. This indicates access to primary dental clinics is within 15 min of driving time in an ideal situation for most primary and secondary schools, which do not account for real-time traffic volumes and any road closures or barriers in the road network. The result also implies that the SDS teams could cover most public primary and secondary schoolchildren within 15 min of drive time. This explains how the SDS team in Selangor could achieve more than 90 per cent coverage including almost all public schools in Selangor over the past 5 years [8].

Although research on the accessibility of dental services is not new in Malaysia, this is the first study to assess the spatial accessibility of schoolchildren in Selangor using travel distance and travel time calculations based on actual road networks. This method has also been used in studies conducted in Australia, the UK, and the US among diverse population groups. All agreed that this method provided a more meaningful representation of spatial access because the road network accounts for geographic barriers, such as rivers, lakes, mountains, and areas without road networks [18,25,26]. In a nutshell, the present study discovered the accessibility of public oral healthcare issues among schoolchildren, the service coverage in terms of driving distance and travel time, and the unequally distributed number of SDS teams across Selangor districts to serve all Selangor schoolchildren. These provide evidence for workforce planning, initiating conversations, and informing decision-makers at the state level about the importance of addressing the apparent misdistribution of the SDS workforce concerning the number of schoolchildren served across the states’ districts. Although the service coverage issues of the SDS in Selangor may not be critical, the study’s findings could highlight a few potential accessibility issues in a rural district in Selangor with a large geographical area. This may generate or widen the inequalities in oral health among schoolchildren in the long term if there is no improvement in the number of facilities in that area [43]. Meanwhile, densely populated areas, such as the *Petaling* district, require the same level of attention due to higher numbers of schools and populations and the need to ensure quality care, despite Selangor’s relatively lower disease burden than other states in Malaysia [8].

Finally, despite the overall improvement in oral health over the last few decades, dental caries and other oral diseases remain extremely common in socially disadvantaged communities, even in an advanced state like Selangor [8]. Therefore, improving access to school dental services for the entire child population, particularly after the pandemic, is critical as the SDS program was temporarily stopped for over 2 years. The evidence provided by this study may assist the SDS team in prioritising the services for those schoolchildren who face barriers in terms of distance and travelling time. Furthermore, dental public health officers or specialists could use the findings to lobby stakeholders to establish new clinics or hospitals to tackle unequal distributions of oral healthcare facilities, redistribute available SDS teams, and appoint newly qualified dental therapists or dentists. As accessibility to healthcare is established as one of the determinants of health, this should be the emphasis in improving the services and, eventually, the oral health and OHRQoL of the targeted population. Geo-mapping the accessibility of schoolchildren in Selangor to public oral healthcare clinics with SDS, therefore, provides evidence that implicates the issues discussed above. At the same time, future research may also explore the association between spatial accessibility and oral health status and dental care utilisation to gain a comprehensive insight into accessibility to oral healthcare among schoolchildren.

## 5. Conclusions

This study addressed the objective and geo-mapped the spatial accessibility of public oral healthcare facilities with SDS mobile teams among public schoolchildren in Selangor regarding travel distance and time from clinics to the schools and vice versa. While the travel distance and time are shorter in urban areas, almost half of the public schools are located more than 5 km from the oral healthcare services and SDS. Most public schoolchildren had access to public oral healthcare within 15–30 min of the driving time of motorized vehicles in ideal traffic situations. However, there are disparities in the distribution of public oral health facilities in urban and rural areas in Selangor. Therefore, primary schools situated more than 20 km from primary dental clinics with SDS, especially in rural districts with large geographical areas, should be prioritized for mobile dental teams or even build new dental facilities. Study findings may be utilized as baseline information in considering the rural–urban gaps in the oral healthcare facilities and the SDS accessibility in Selangor. In addition, there is a continuing need to maintain the SDS program via outreach mode conducted by the mobile dental team, especially in rural areas. Geo-mapping highlights the inequalities in spatial accessibility to public oral health facilities with SDS among schoolchildren in Selangor. It is time to prioritise the resources, SDS, and preventive programmes to reduce inequalities in oral health accessibility among schoolchildren in Selangor.

## Figures and Tables

**Table 1:** Main Administrative Districts, Location Strata, and Mean Household Income in Selangor.

District	Location Strata	Mean Household Income	Total Districts
Gombak	Urban	11,536	
Klang	Urban	9980	
Kuala Selangor	Rural	7866	
Kuala Langat	Rural	8375	
Petaling	Urban	12,145	
Sabak Bernam	Rural	6042	
Sepang	Urban	12,254	
Hulu Langat	Urban	10,252	
Hulu Selangor	Rural	7598	
Selangor			9

**Table 2:** Public oral health facilities in Selangor.

	Dental Clinic			Total Primary Care Clinic	Hospital-Based Clinic	Non-Hospital-Based Clinic			Total
Districts	Primary Clinic	Visiting	UTC			Orthodontic	Periodontic	Restorative	
Gombak	8	2	0	10	2	1 *	0	1	
Klang	7	1	0	8	1	2	1 *	1	
Kuala Selangor	3	2	0	5	0	1	0	0	
Kuala Langat	3	5	0	8	0	0	0	0	
Petaling	7	0	1	8	1	2	1	1	
Sabak Bernam	4	3	0	7	0	0	1	0	
Sepang	2	1	0	3	1	0	0	0	
Hulu Langat	11	0	0	11	2	1	1	1	
Hulu Selangor	3	4	0	7	0	0	0	0	
Total	48	18	1	67	7	7	4	4	89

* Standalone non hospital-based clinic. Others (13 non-hospital based) are in the same compound with primary oral health facilities.

**Table 3:** Travel distance of primary schools outside 5 km, 10 km, and 20 km service area.

District	Location Strata	Primary Dental Clinics	SDS Mobile Dental Team	Number of Schools	Number of Primary Schoolchildren	Schools Outside 5 km	Primary Schoolchildren Outside 5 km	Percentage of Schools Outside 5 km	Percentage of Schoolchildren Outside 5 km	Schools Outside 10 km	Primary Schoolchildren Outside 10 km	Percentage of Schools Outside 10 km	Percentage of Schoolchildren Outside 10 km	Schools Outside 20 km	Primary Schoolchildren Outside 20 km	Percentage of Schools Outside 20 km	Percentage of Schoolchildren Outside 20 km
Gombak	Urban	10	9 *	70	70,405	22	19,131	31.43	27.17	10	10,633	14.29	15.10	0	0	0.00	0.00
Klang	Urban	8	7 *	94	91,442	31	33,997	32.98	37.18	6	4057	6.38	4.44	3	363	3.19	0.00
Kuala Selangor	Rural	8	5 *	71	28,181	55	22,008	77.46	78.10	31	16,020	43.66	56.85	5	149	7.04	0.02
Kuala Langat	Rural	8	5 *	56	27,851	39	19,489	69.64	69.98	3	3472	5.36	12.47	2	80	3.57	0.01
Petaling	Urban	7	6 *	147	149,031	68	63,389	46.26	42.53	4	4599	2.72	3.09	0	0	0.00	0.00
Sabak Bernam	Rural	7	4 *	48	10,916	27	3712	56.25	34.01	11	2678	22.92	24.53	0	0	0.00	0.00
Sepang	Urban	3	3 *	40	24,311	27	14,619	67.50	60.13	12	2507	30.00	10.31	0	0	0.00	0.00
Hulu Langat	Urban	11	5 *	91	99,098	20	18,319	21.98	18.49	0	0	0.00	0.00	0	0	0.00	0.00
Hulu Selangor	Rural	7	5 *	50	23,641	35	16,965	70.00	71.76	26	13,003	52.00	55.00	1	7	2.00	0.01
Selangor		67	49	667	524,876	324	211,629	52.61	48.82	103	56,969	19.70	20.20	11	599	1.76	0.01

* SDS Mobile dental team stationed in Primary Dental Clinics.

**Table 4:** Travel distance of secondary schools outside 5 km, 10 km, and 20 km service area.

District	Location Strata	Primary Dental Clinics	SDS Mobile Dental Team	Number of Schools	Number of Secondary Schoolchildren	Schools Outside 5 km	Secondary Schoolchildren Outside 5 km	Percentage of Schools Outside 5 km	Percentage of Schoolchildren Outside 5 km	Schools Outside 10 km	Secondary Schoolchildren Outside 10 km	Percentage of Schools Outside 10 km	Percentage of Schoolchildren Outside 10 km	Schools Outside 20 km	Secondary Schoolchildren Outside 20 km	Percentage of Schools Outside 20 km	Percentage of Schoolchildren Outside 20 km
Gombak	Urban	10	9 *	34	47,761	7	12,496	20.59	26.16	5	5869	14.71	46.97	0	0	0.00	0.00
Klang	Urban	8	7 *	38	55,868	10	18,130	26.32	32.45	2	1502	5.26	8.28	1	359	2.63	0.64
Kuala Selangor	Rural	8	5 *	19	19,954	7	9766	36.84	48.94	6	9006	31.58	92.22	0	0	0.00	0.00
Kuala Langat	Rural	8	5 *	18	19,270	2	10,683	11.11	55.44	4	4574	22.22	42.82	0	0	0.00	0.00
Petaling	Urban	7	6 *	73	90,915	45	50,793	61.64	55.87	2	2626	2.74	5.17	0	0	0.00	0.00
Sabak Bernam	Rural	7	4 *	18	11,909	8	50,017	44.44	419.99	7	4068	38.89	8.13	1	535	5.56	4.49
Sepang	Urban	3	3 *	12	14,399	6	9611	50.00	66.75	2	2542	16.67	26.45	0	0	0.00	0.00
Hulu Langat	Urban	11	5 *	39	63,350	4	8219	10.26	12.97	0	0	0.00	0.00	0	0	0.00	0.00
Hulu Selangor	Rural	7	5 *	18	17,447	11	10,117	61.11	57.99	8	7960	44.44	78.68	1	276	5.56	1.58
Selangor		67	49	269	340,873	107	180,462	39.78	52.94	36	38,147	13.38	21.14	3	1170	1.12	0.34

* SDS Mobile dental team stationed in Primary Dental Clinics.

**Table 5:** Travel-time of primary and secondary schools outside 15 min and 30 minutes’ drive time.

Districts	Location Strata	No. of Primary Schools Outside 15 min Travel Time	No. of Primary Schoolchildren Students	Percentage of Schools within 15 min	Percentage of Schoolchildren within 15 min	Percentage of Schools Outside 15 min	Percentage of Schoolchildren Outside 15 min	No. of Secondary Schools Outside 15 min Travel Time	No. of Primary Schools Outside 30 min	Percentage of Secondary Schools within 15 min	Percentage of Schoolchildren within 15 min	No. of Primary Schools Outside 30 min Travel Time	No. of Primary School Students	Percentage of Primary Schools Outside 30 min	Percentage of Schoolchildren Outside 30 min	No. of Secondary Schools Outside 30 min Travel Time	No. of Secondary Schools Students	Percentage of Primary Schools Outside 30 min	Percentage of Primary Schoolchildren Outside 30 min
Gombak	Urban	0	0	100.00	100.00	0.00	0.00	2	3493	5.88	7.31	0	0	0	0	0	0	0.00	0.00
Klang	Urban	3	363	96.81	99.60	3.19	0.40	1	359	2.63	0.64	1	363	0	0	0	0	0.00	0.00
Kuala Selangor	Rural	5	169	92.96	99.40	7.04	0.60	0	0	0.00	0.00	1	76	1.41	0.27	1	359	5.26	1.80
Kuala Langat	Rural	2	209	96.43	99.25	3.57	0.75	0	0	0.00	0.00	1	43	1.79	0.15	0	0	0.00	0.00
Petaling	Urban	0	0	100.00	100.00	0.00	0.00	0	0	0.00	0.00	0	0	0.68	0.00	0	0	0.00	0.00
Sabak Bernam	Rural	0	0	100.00	100.00	0.00	0.00	2	796	11.11	6.68	0	0	2.08	0.00	1	535	5.56	4.49
Sepang	Urban	0	0	100.00	100.00	0.00	0.00	0	0	0.00	0.00	0	0	2.50	0.00	0	0	0.00	0.00
Hulu Langat	Urban	2	439	97.80	99.56	2.20	0.44	0	0	0.00	0.00	0	0	1.10	0.00	0	0	0.00	0.00
Hulu Selangor	Rural	9	5414	82.00	77.10	18.00	22.90	2	2162	11.11	12.39	0	0	2.00	0.00	0	0	0.00	0.00
Selangor		23	6594	96.55	98.74	3.45	1.26	7	6810	2.60	2.00	8	482	0.15	0.09	2	894	0.74	0.26

## Data Availability

Not applicable.
